# “In the United States, we say, ‘No breastfeeding,’ but that is no longer realistic*”*: provider perspectives towards infant feeding among women living with HIV in the United States

**DOI:** 10.1002/jia2.25224

**Published:** 2019-01-18

**Authors:** Emily L Tuthill, Cecilia Tomori, Meredith Van Natta, Jenell S Coleman

**Affiliations:** ^1^ Department of Community Health Systems School of Nursing University of California San Francisco CA USA; ^2^ Department of Anthropology Durham University Durham UK; ^3^ Department of Epidemiology Johns Hopkins Bloomberg School of Public Health Baltimore MD USA; ^4^ Department of Social and Behavioral Sciences University of California San Francisco CA USA; ^5^ Department of Gynecology/Obstetrics Johns Hopkins University School of Medicine Baltimore MD USA

**Keywords:** breastfeeding, HIV, United States, harm reduction, provider attitudes, infant feeding

## Abstract

**Introduction:**

Currently, the United States (U.S.) recommends that infants born to women living with HIV (WLHIV) be fed formula, whereas many low‐resource settings follow the World Health Organization's recommendation to exclusively breastfeed with ongoing antiretroviral therapy. Evidence on infant feeding among WLHIV in high‐resource countries suggest that these contrasting recommendations create challenges for providers and patients. Our study used multiple methods to understand providers’ infant feeding perspectives on caring for their pregnant and post‐partum WLHIV in the U.S.

**Methods:**

We sent a survey (n = 93) to providers across the U.S. who have cared for WLHIV. A subset of survey participants opted into a follow‐up qualitative interview (n = 21). These methods allowed us to capture a broad understanding of provider attitudes via the survey and more nuanced qualitative interviews. The study was completed prior to an updated breastfeeding section of the U.S. Perinatal Guidelines.

**Results:**

The majority of providers (66.7%) discussed infant feeding intent with their patients using open‐ended questions. Many also discussed alternative feeding methods (37.6%) and disclosure avoidance strategies (34.4%). Over 75% (95% confidence interval (CI): 65.1 to 84.2) of participants reported that a WLHIV asked if she could breastfeed her child, and 29% (95% CI 20 to 40.3) reported caring for a patient who breastfed despite recommendations against breastfeeding. Providers reported that their patients’ primary concern was stigma associated with not breastfeeding (58%), while providers were primarily concerned about medication adherence during breastfeeding (70%). Through qualitative analysis, four overarching categories emerged that reflect providers’ sentiments, including (1) U.S. guidelines inadequately addressing WLHIV's desire to breastfeed; (2) negotiating patient autonomy amidst complex feeding situations; (3) harm reduction approaches to supporting WLHIV in breastfeeding; and (4) providers anticipating multilayered patient stigmatization.

**Conclusions:**

The majority of provider respondents cared for a WLHIV who desired to breastfeed, and a third had WLHIV who breastfed despite recommendations against it. Providers found that the status of U.S. guidelines and their incongruity with WHO guidelines left them without adequate resources to support WLHIV's infant feeding decisions. Our findings provide important insight to inform professional associations’ discussions about public health policy as they consider future directions for infant feeding guidelines among WLHIV.

## 1 Introduction

The American Academy of Pediatrics and the United States (U.S.) Department of Health and Human Services currently recommend women living with HIV (WLHIV) in the U.S. to exclusively formula feed their infants due to the risk of HIV transmission through breast milk 1, 2, 3. Several studies from low‐resource settings demonstrate low rates of HIV transmission (0% to 3%) with antiretroviral therapy (ART) use before and during breastfeeding 4, 5, 6, 7, 8, 9, 10, 11, 12, 13. In these low‐resource settings where diarrhoeal diseases, pneumonia and malnutrition are common, access to clean water may be limited and infant formula may be expensive or inaccessible, the World Health Organization (WHO) recommends exclusive breastfeeding for six months and continued breastfeeding up to 24 months with ongoing ART use by mother and infant 14. In these settings, breastfeeding decreases risks of infant morbidity and mortality 15, 16.

The contrast in recommendations between low‐resource and high‐resource settings has created challenges for providers and patients in the U.S. and other high‐resource settings 17, 18, 19, 20, 21, 22, 23, 24. Indeed, in these high‐resource settings, national public health organizations promote exclusive breastfeeding and continued breastfeeding up to two years among women who are HIV negative. Although there is limited research from high‐resource settings, investigating perceptions of infant feeding among WLHIV, three qualitative studies (one from the United Kingdom 20 and two from Canada 22, 23) illustrate common sentiments. WLHIV reported feeling like they were not fulfilling their role as mothers 20, 23. Many women perceived they were disclosing their HIV status by formula feeding given they were often asked by friends, family and community members why they were not breastfeeding. In addition, women felt guilt, shame and stigma for not being able to breastfeed their infants, in addition to practical difficulties such as affording, procuring and preparing infant formula 20.

Consequently, stakeholders within the field of prevention of mother to child transmission of HIV, including researchers, providers and patients, have raised questions over the ethics of maintaining disparate guidelines, warning that this may hinder patient–provider communication and overlook the experiences of their patients who have immigrated to the U.S. 21, 22. Levison *et al*. further suggest that WLHIV in the U.S. may desire to breastfeed because of the emotional, cultural and/or nutritional benefits of breastfeeding, and they suggest a harm reduction approach for counselling these women 24. Recently, the British Perinatal Guidelines and U.S. Perinatal Guidelines contained updates that offer a new section providing some guidance for providers with patients who wish to breastfeed 3, 25. Importantly, the updated guidelines continue to endorse exclusive formula feeding and emphasize their recommendation to avoid breastfeeding. However, in the event that a WLHIV has received extensive counselling and plans to breastfeed, a harm reduction approach is recommended. This strategy includes recommendations for HIV treatment and adherence monitoring, breastfeeding duration and weaning, and HIV treatment prophylaxis recommendations for infants. Although these recommendations provide official guidance for providers, gaps remain.

Providers are encountering these infant feeding dilemmas more often in their practices; however, there is insufficient evidence on their perspectives and current experiences. To fill these gaps, our study employed a multiple methods approach to better understand U.S. providers’ attitudes towards and experiences of caring for WLHIV, and how they navigate their patients’ infant feeding expectations.

## 2 Methods

An online survey was created that included two sections: twenty‐eight closed‐ended items and eight open‐ended questions. It was pilot tested among 10 providers. The survey asked questions regarding attitudes towards infant feeding and counselling approaches – both in general and specifically for WLHIV (see Tables 1 and 2). The anonymous survey was emailed weekly for one month in 2016 to providers who subscribed to a reproductive infectious disease listserv. The listserv had 367 active provider email addresses from several countries. The listserv is an international forum to connect clinicians and non‐clinicians caring for perinatal WLHIV to discuss difficult cases, share tools, protocols, ask questions. Only U.S. providers were asked to participate. Participants who completed the survey could then opt in to doing a semi‐structured interview.

**Table 1:** Demographics and employment characteristics of survey participants by response^a^

	Total (N = 93)	Would not offer BF	Would offer BF or uncertain	*p*‐value
Age category (years)^b^				
<40	34 (37)	2 (13.3)	26 (40.6)	0.06
40 to 49	26 (28.3)	4 (26.7)	19 (29.7)	
≥50	32 (34.8)	9 (60)	19 (29.7)	
Race/ethnicity				
Non‐Hispanic White	61 (65.6)	10 (66.7)	42 (33.3)	0.50
Non‐Hispanic non‐White	26 (28)	5 (33.3)	17 (26.6)	
Mixed race	6 (6.5)	0 (0)	5 (7.8)	
Sex				
Female	82 (88.2)	11 (73.3)	57 (89.1)	0.11
Male	11 (11.8)	4 (26.7)	7 (10.9)	
Professional degree				
MD	64 (68.8)	14 (93.3)	42 (65.6)	0.21
Advanced practice provider	25 (26.9)	1 (6.7)	19 (29.7)	
Social work	2 (2.2)	0 (0)	2 (3.1)	
Registered nurse	2 (2.2)	0 (0)	1 (1.6)	
Years in practice, median (IQR)	12 (5 to 23)	21 (10 to 26)	12 (4 to 20.5)	0.04
Practice setting				0.52
Academic	64 (68.8)	11 (73.3)	48 (75)	
Community	24 (25.8)	3 (20)	15 (23.4)	
Government or other	5 (5.4)	1 (6.7)	1 (1.6)	
Clinical role				
OB/GYN	42 (45.1)	7 (46.7)	27 (42.1)	0.81
Adult infectious diseases or primary care	36 (38.7)	5 (33.3)	27 (42.1)	
Paediatric infectious diseases or primary care	15 (16.1)	3 (20)	10 (15.6)	
U.S. Location				
North‐east	20 (21.5)	4 (26.7)	12 (18.8)	0.74
Midwest	12 (12.9)	1 (6.7)	10 (15.6)	
South‐east	33 (35.5)	6 (40)	22 (34.4)	
West	28 (30.1)	4 (26.7)	20 (31.3)	

BF: breastfeeding; IQR: interquartile range. ^a^Survey Question: In African clinical trials, the risk of mother to child transmission is less than 2% with infant HIV prophylaxis and an undetectable viral load in mothers taking highly active antiretroviral medication. If the patient was willing to accept the risk, would you consider offering breastfeeding as an option? Reponses are n(%) unless otherwise stated; ^b^age is missing one response.

**Table 2:** Responses to survey questions^a^

**Providers’ approach to counselling**	**N = 93**
***When you bring up the topic of breastfeeding in women living with HIV do you:***	n *(%)*
Acknowledge the recommendation against breastfeeding, but begin an open‐ended discussion with patients to find out more about their desires to breastfeed	62 (66.7)
Discuss alternatives to formula feeding with your patients (e.g. using banked human breast milk)	35 (37.6)
Discuss options that might give the appearance of breastfeeding if your patients are concerned that not being able to breastfeed will reveal their HIV status	32 (34.4)
Provide counselling and support to assist women living with HIV with breastfeeding	20 (21.5)
Tell women living with HIV that they cannot breastfeed without further discussion	15 (16.1)
**Open‐ended responses**	
*“I recommend against breastfeeding, but do engage them in conversation about it*.*”*	
*“Will work with a woman if she is determined to breastfeed”*	
*“I acknowledge the issue of post‐partum feeding and that breast feeding is not encouraged due to HIV transmission risk to the baby.”*	
*“Discuss the possible risk of breast feeding and supplemental formula feeding on infant transmission risk”*	
*“Discuss challenges regarding outside caregivers or breast problems; infant bonding techniques without breastfeeding”*	
*“Explain risk with breastfeeding, review bottle feeding”*	
*“I don't discuss BF as an equal alternative to formula, but do review the rationale/context”*	
*“Tell her in this country because of access to clean water, formula feeding is recommended, but acknowledge that women in Africa on ART do breastfeed. Also that chances of transmission are hugest for inexperienced first time nursing moms. This was to an African woman who had migrated here and had breast fed all her other kids. I was not sure what she would do once she delivered, as I felt she may decide to breastfeed despite the U.S. recommendations and wanted her to have the full information. She was suppressed and the chances of transmission were likely extremely low, especially past the first day or two. If she had chosen to breast feed I would have supported her. She chose not to. Told her housemates that her doctor had told her a medicine the doctor gave meant she could not breast feed and they accepted that.”*	
*“Provide options and resources!”*	
*“Explain recommendation and current understanding about [viral load] in breast milk and encourage options and troubleshoot cultural attitudes and expectations”*	
**Patients’ concerns about not breastfeeding**	
***What concerns do*** ***your patients*** ***living with HIV raise about not being able to breastfeed?***	
Not being able to reap the health benefits of breastfeeding for mother and/or infant	47 (50.5)
Cost of formula feeding	13 (14)
Stigma associated with not breastfeeding with family/community	54 (58.1)
Stigma associated with formula feeding in society	15 (16.1)
Physical discomfort of breast engorgement	9 (9.7)
Not being able to bond with their child through breastfeeding	35 (37.6)
None of these	4 (4.3)
**Open‐ended responses:**	
*“They always seem to already know it is not recommended”*	
**Providers’ concerns about supporting breastfeeding**	
***What concerns do*** ***you*** ***have about supporting your HIV positive patients in breastfeeding?***	
The ongoing risk of mother to child transmission	61 (65.6)
Concern for non‐compliance with ARV and/or extended infant prophylaxis	65 (70)
Concern for side effects of extended infant prophylaxis	32 (34.4)
Legal risks associated with potential transmission	21 (22.6)
**Open‐ended responses:**	
*“Mixed breast + bottle feeding”*	
*“The legal concern is something that I don't think should enter one's decision, when one is going right by the patient based on the available data. It would be nice to have back up in some kind of statement from a society/guideline group, more to appease horrified colleagues.”*	
*“Discordance of viral load in other compartments such as breastmilk. The small risk of transmission when there are very safe alternatives in the US for infant feeding which is not the case internationally*.*”*	
*“Difficulty for mother to manage infant prophylaxis”*	
**Type of support needed to support breastfeeding**	
***What mechanisms would have to be in place for you to assist your HIV positive patients with a consistently undetectable viral load with breastfeeding?***	
None. I probably would never assist an HIV positive mother with breastfeeding	7 (7.5)
Clinical guidelines (i.e. government)	58 (62.4)
Committee opinion developed by a national organization (i.e. ACOG, AAP, IAS)	59 (63.4)
Legal protection	25 (26.9)
Clinical data on benefits	48 (51.6)
**Open‐ended responses:**	
*“If they decide to breastfeed that is their prerogative and I would assist them despite disagreeing with them”*	
*“Patient preference”*	
*“Only if living in resource poor country”*	
*“Data on real world practice (not just from clinical trials where there is a specific program for providing access to care and long term monitoring with a lot of resources of study personnel and study funds.”*	
*“Team approach with a supportive pediatrician”*	
*“We developed a detailed counseling guide which we review with mom and ask her to sign acknowledging risk and understanding. We then place infant on ARV throughout breastfeeding, monitor mother and infant monthly for HIV viral load and ARV toxicity, and advocate for rapid weaning. It has been very stressful on clinicians and mothers alike, and lots of work*.*”*	
*“Data that the benefits of breastfeeding really do outweigh the transmission risk*.*”*	
*“Again would try to prevent breastfeeding in US as I believe it medically wrong to expose the baby to any risk of HIV transmission where none exists*.*”*	
*“The more of these that are in place, the easier to convince providers to be more permissive with patients*.”	

ART, antiretroviral therapy. ^a^Participants could select more than one answer

ET and CT conducted semi‐structured interviews with participants either in person or by phone. Interviews were recorded, transcribed and ranged from 22 to 61 minutes (median 38 minutes). Interview content related to providers’ approaches to counselling WLHIV about infant feeding, their knowledge of and attitude towards national and international infant feeding guidelines, and concerns they or their patients had (if any) around HIV and infant feeding. Data collection for the survey and qualitative interviews occurred between September 2016 through April 2017.

An institutional review board approval was granted at Johns Hopkins University and the University of California San Francisco. The completion of the survey served as consent, and an oral consent process was obtained for the semi‐structured interview. Interviewed participants were offered a $25 electronic Amazon^®^ gift card for their time.

## 3 Data analysis

### 3.1 Survey

Demographic data, practice characteristics and closed‐ended survey responses were summarized. Percentages with 95% confidence intervals (CIs) were calculated. A chi‐squared or Fisher's exact test was performed to compare categorical data. Some survey questions included a write‐in option when “other” was selected as a response. These responses were included verbatim in quotations. STATA (College Station, Texas version 15) was used.

### 3.2 Qualitative semi‐structured interviews

We followed a qualitative content analysis approach 26 to systematically code and classify the data into categories. Transcripts were entered and coded in Dedoose (version 7.6.21). We began with emergent coding and segmentation of a random selection of one third of the data (seven transcripts). The basic unit of analysis was segments of the providers’ descriptions of their experiences with and/or responses to WLHIV breastfeeding, including opportunities and challenges they have encountered. We then considered the appropriateness and reliability of the emerging codes and developed a preliminary codebook before resuming inductive coding of the remaining transcripts. Frequent codes, and codes with high rates of co‐occurrence, were exported to Word for closer analysis and conceptual data clustering. These included initial clusters such as “reducing harm,” “coordinating care” and “stigma/disclosure”. Using subsumption and progressive summarizing 27, the team began classifying clusters into broad categories. These categories and subcategories were then depicted as dendrograms.

## 4 Results

### 4.1 Quantitative survey

One hundred and eight providers began the survey; 15 were excluded because they lived outside the U.S. or did not provide care to WLHIV or their children. A total of 93 respondents were eligible for inclusion in analysis. The majority of respondents were non‐Hispanic White women (Table 1). Over two‐thirds practised in an academic setting. Obstetrics/gynecology (OB/GYN) was the most common specialty (45.1%), followed by adult medicine (e.g. infectious diseases, HIV medicine, primary care; 38.7%) and pediatric medicine (16.1%).

The majority of providers (66.7%) reported discussing infant feeding intention with their patients using open‐ended questions (Table 2). Many also discussed alternative feeding methods (37.6%) and strategies to avoid disclosure of their HIV status by giving the appearance of breastfeeding (34.4%). Some providers (21.5%) offered additional counselling and support to assist WLHIV with breastfeeding, while 16.1% reported telling WLHIV that they cannot breastfeed without further discussion. Over 75% (95% CI: 65.1 to 84.2) reported that a WLHIV asked if she could breastfeed her child, and 29% (95% CI: 20 to 40.3) reported caring for a WLHIV patient who breastfed despite receiving recommendations to not breastfeed. Providers reported that approximately half of those WLHIV who asked to breastfeed and who breastfed despite recommendations were immigrants. From the provider perspective, the most frequently cited concerns of their patients included stigma from family or their community from not breastfeeding (58%) and not reaping the health benefits of breastfeeding for their infants (50.5%). The most frequently cited concerns of providers included medication non‐adherence (70%) and the ongoing risk of mother‐to‐child HIV transmission with breastfeeding (65.6%).

### 4.2 Qualitative interviews

Of the 93 survey participants, 21 (23%) completed the optional semi‐structured interview. Participants in the semi‐structured interview included two pediatricians; six OB/GYN physicians, nurses and midwives; two social workers; one nurse practitioner, one infectious disease specialist and one HIV/family practice specialist. Of the remaining eight participants, seven were physicians who did not specify their clinical specialty. Participants had varying degrees of clinical experience working with mothers living with HIV, ranging from only a few such patients to the majority of their practice. Participants covered 11 states across all regions of the U.S. Two‐thirds of participants worked in an academic setting at the time of the interview, and the remaining third worked in a community setting. About two‐thirds of participants were over 40 years old, and 70% were female.

Four main qualitative categories emerged (Box 1), including (1) perception of U.S. guidelines as inadequately addressing WLHIV's desire to breastfeed; (2) providers negotiating patient autonomy amidst complex feeding situations; (3) harm reduction approaches for supporting WLHIV in breastfeeding; and (4) providers anticipating multilayered patient stigmatization.

Box 1 **Illustrative Quotes from the Qualitative Semi‐structured Interviews**

**Category 1: Perception of U.S. guidelines as inadequately addressing WLHIV's desire to breastfeed**

…it's becoming more clear that you can't just say … this continent breastfeeds, these other continents don't breastfeed. Physician

It's this irrational, surreal thing… that women, when they hit the ground in Liberia, they're supposed to start breastfeeding, but while they're here [in the U.S.], they're not. Pediatric HIV specialist

I recently came back from Malawi, they kind of roll their eyes when I tell them this is even an issue in the U.S. because there, exclusive breastfeeding, it's the norm and that's the standard of care. Obstetrician

**Category 2: Negotiating patient autonomy amidst complex feeding situations**

I tell the patients at the beginning that they have to have some trust that I will do the best for them and for the infant. … They asked me to fly this plane for them. Obstetrician

[Patients] are coming to these questions based on just the fact that they're living for a long time, and they're taking meds, and the meds are working, and so why wouldn't breastfeeding be an option? Nurse practitioner

I always acknowledge with women how hard it is, if you want to breastfeed, not to breastfeed. Midwife

I did feel badly because if really the risks are extremely low and the person is suffering in some way mentally, psychologically by not [breastfeeding]… Physician

**Category 3: Harm reduction approaches for supporting WLHIV in breastfeeding**

… the main concern would be that a mother would choose to do this and not feel like she could talk to her provider about it, so then there'd be no way of actually helping to offer more support and monitoring in that situation. Nurse practitioner

…we had a really good team approach… We made sure, postpartum, to touch base with the care team that delivered and the nurses, in‐patient, to make sure they knew no formula, no formula, no formula. Nurse

Have a long history of undetectable viral load, certainly throughout pregnancy, but beyond that, probably even knowing that they would have ongoing insurance coverage for the mom to be able to still maintain their medication. OB RN

I would want to keep her in our multidisciplinary perinatal clinic as long as she was breastfeeding… so she can continue to benefit from those resources and make sure that we're doing all the things we did during pregnancy to keep her viral loads suppressed. Obstetrician

**Category 4: Providers anticipating multilayered patient stigmatization**

If you're American born, you have been cultured for the most part that breastfeeding is an option. And if you don't choose it, you're not choosing it for a bunch of reasons. Where my African born or Caribbean born women, if you don't breastfeed… it's because you have HIV. Like one equals the other. Unspecified provider

I usually try to suggest some things that they could say to why they're not [breastfeeding], so you're on blood pressure medication, or you're on diabetes medicine, or things like that to help. Nurse practitioner

HIV is stigmatizing, and you see everywhere, if you have any access to any form of media, that breast is best, breast is best… It's like you hear it in your head, and then you have these women who are told, ‘Your body actually isn't best.’ So, that's even further stigmatizing… Nurse

### 4.3 Category 1: Perception of U.S. guidelines as inadequately addressing WLHIV's desire to breastfeed

Of note, the study was done prior to the update in the U.S. Perinatal Guidelines. Many providers acknowledged that current U.S. guidelines were inadequate in circumstances where their patients desired to breastfeed. While some providers adhered to what they believed were insufficient guidelines, others described developing provisional protocols to handle requests to breastfeed. Some considered other HIV risk scenarios, such as embracing pre‐exposure prophylaxis or vaginal delivery, to rationalize a more proactive approach to supporting WLHIV to breastfeed.

Providers also recognized the paradox of divergent guidelines in globalized societies and expressed frustration that these discrepant guidelines did not meet patients’ needs. Many were aware of studies in low‐resource countries that indicated low rates of HIV transmission with exclusive breastfeeding, and often their patients who had emigrated from these countries had personal experience with distinct feeding recommendations in their home countries. These patients’ lives spanned multiple continents, and many were uncertain whether they would return post‐partum to their home countries, posing a dilemma for infant feeding decisions.

### 4.4 Category 2: Negotiating patient autonomy amidst complex feeding situations

A second content category involved providers negotiating patient autonomy within complex feeding situations. Providers described a range of patient discomfort with the zero‐breastfeeding guideline and/or ability to follow it. This meant they had to assess patients’ stated priorities alongside their psychological wellbeing and potential structural barriers to formula feeding. While some providers served populations for which formula feeding was common practice, many encountered situations requiring more detailed discussion of feeding practices. Interviews revealed a continuum wherein a few providers described taking a firm lead to guide patients towards formula feeding, while many others prioritized patients’ autonomy. Providers also reported that while some patients wanted them to make the risk‐benefit calculations on their behalf, others (particularly immigrant patients or U.S.‐born, English‐speaking mothers who had lived with HIV for many years) stressed the benefits of breastfeeding despite receiving recommendations from health professionals to formula feed.

Even among mothers who agreed to forgo breastfeeding, providers identified psychological and structural challenges around formula feeding that required careful negotiation. This was particularly true in cases of immigrant mothers. Some providers expressed concern that prohibiting a patient from breastfeeding would lead to psychological distress, whether by impeding a mother's cultural norms or her sense of bonding with her infant. Other providers stressed more structural concerns related to formula feeding. For instance, navigating Woman, Infants and Children (WIC) benefits and accessing formula feeding education and support was challenging for women who were unfamiliar with formula feeding and/or using bottles. Thus, mothers who follow U.S. guidelines by deciding *not* to breastfeed may also require ongoing psychological and social support to carry out that decision.

### 4.5 Category 3: Harm reduction approaches for supporting WLHIV in breastfeeding

**Figure 1:**
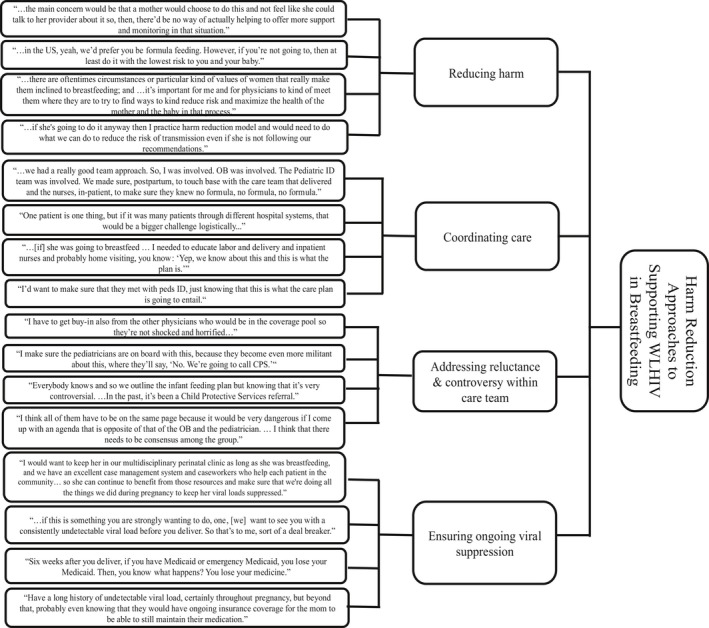
Dendogram of category 3: Harm Reduction Approaches to Supporting women living with HIV (WLHIV) in Breastfeeding

Harm reduction emerged as a common strategy among several providers when facing infant feeding dilemmas, which is illustrated in Figure 1. While few providers promoted developing their own protocols that differed from governing bodies’ recommendations, most providers acknowledged that if a WLHIV was determined to breastfeed, they needed to help minimize risks. These providers recounted various approaches to reducing harm in such situations, including coordinating care across health providers and systems, addressing resistance within the care team and ensuring ongoing viral suppression.

Ensuring the success of harm reduction meant mobilizing the entire team, including providers within obstetrics, pediatrics and social work to agree on a shared care plan. This helped to avoid escalations such as reporting breastfeeding events to child protection agencies. Other harm reduction strategies included practitioners seeking out information about the context in which women would be executing infant feeding plans, including whether a woman planned to stay in the U.S. or attitudes within communities on infant feeding behaviours. For example, WLHIV returning to their home countries might encounter the insecurity of formula feeding abroad, while those who planned to remain within an immigrant community might face pressures to breastfeed when among family, friends and housemates.

### 4.6 Category 4: Providers anticipating multilayered patient stigmatization

A final category highlights providers’ awareness of the multiple, intersecting layers of stigma facing pregnant and post‐partum WLHIV. This included both the association of formula feeding with HIV in some communities, which could lead to unwanted disclosure and HIV‐related stigmatization, and the stigmatization of formula feeding in environments where breastfeeding has become the social norm. In the former case, some providers attempted to support WLHIV to formula feed by suggesting an alternative narrative. This strategy was most commonly used among immigrants from cultures where breastfeeding was the cultural norm and formula feeding was interpreted as a sign of HIV. In the latter case, the recent push in many U.S. institutions to promote breastfeeding has contributed additional stigma among WLHIV who are formula feeding their child. One provider channelled how these multiple layers of stigma became internalized by patients: *“*Well, I already feel like a bad person because I have HIV. Now I feel like a bad person because I have HIV and I'm pregnant. And now a really bad person because I can't breastfeed my baby.*”*

## 5 Discussion

Using multiple methods of inquiry, we contextualized the infant feeding environment as experienced by providers’ perspectives from caring for WLHIV or their infants in the U.S. The majority of provider respondents had a WLHIV at their practice ask if she could breastfeed, and a third reported that a WLHIV breastfed despite the recommendation to formula feed. We found that providers recognized that their patients faced complex infant feeding situations, and they expressed concern that the current formula‐only guidelines did not serve the full range of their clinical needs. Our study was conducted prior to the recent update of the U.S. Perinatal Guidelines 3, and thus reflected the dilemma providers faced at the time of the interview. Despite this, providers described the range of harm reduction strategies they undertook to manage complex infant feeding situations. Several providers encountered patients who resisted the recommendation due to personal priorities and/or social barriers that the guidelines did not address. They described their concerns about facilitating adherence to current guidelines in the light of patients’ experiences of multiple, compounding layers of stigmatization associated with formula feeding and HIV, and the potential for unwanted disclosure of HIV status due to formula feeding. Providers’ desire to respect mothers’ autonomy further complicated their ability to adhere to a zero‐breastfeeding guideline. In response to their patients’ needs, many providers adopted a harm reduction philosophy stressing formula feeding as the best practice while acknowledging that some patients could not, or would not, follow it. Others continued to follow the guidelines to the letter, with varying levels of patient and provider satisfaction.

Previous findings from Canada, the United Kingdom and the U.S. indicate that conflicting global guidelines and patients’ and providers’ awareness of research on HIV transmission with ART has contributed to concerns about recommendations against breastfeeding. Our study is the first in the U.S. to voice providers’ perspectives and explore their current experiences on WLHIV breastfeeding. Migration both to and from the U.S. presented the most acute challenges for many providers and their patients. While providers shared that some patients expressed opposition to formula feeding on the basis that they perceived breastfeeding as superior to formula feeding in terms of nutrition and/or bonding, providers more frequently attributed patient resistance to situations in which a combination of women's culture, precarious legal status and disclosure danger made formula feeding a risky choice for them. Clinical guidelines must consider obstacles to formula feeding among women who migrate from low‐resource to high‐resource countries. While providers are primarily concerned about cultural barriers to formula feeding in immigrant communities, immigrant WLHIV also face numerous structural challenges, including uncertainties in U.S. immigration policy and ineligibility and/or lack of access to affordable medical care and public benefits such as WIC. Moreover, women may return to their home countries and therefore may be unable to adhere to U.S. guidelines. These issues may continue to present challenges for providers despite the update in U.S. Perinatal Guidelines 3.

The survey revealed that 70% of provider respondents were concerned about post‐partum ART adherence, although this emerged only as a secondary finding in the semi‐structured interviews. Several retrospective cohort U.S. studies demonstrate poor post‐partum retention in care and viral suppression 28, 29, 30, which represents real‐world scenarios in contrast to the highly supported and well‐resourced breastfeeding clinical trials. The WHO acknowledges this concern and advises skilled counselling and adherence support for post‐partum women 14. In settings where guidelines recommend exclusively breastfeeding for six months and continued breastfeeding up to twenty‐four months with ongoing maternal and infant ART use, the WHO states health authorities should actively coordinate and implement services in health facilities and activities in workplaces, communities and homes to protect, promote and support breastfeeding among WLHIV. Much of this has been initiated in prevention of mother to child transmission clinic services. However, operationalizing this statement in the U.S. may pose challenging given suboptimal engagement in care post‐partum among all U.S. women 31.

### 5.1 Limitations

This study aimed to gain insight into providers’ attitudes of infant feeding caring for pregnant and post‐partum WLHIV, but the small survey sample limited statistical power and made it difficult to assess associations. Therefore, we were only able to present descriptive data. Additionally, the sample overrepresented academic providers and unevenly represented the variety of clinical roles, which might have led to selection bias. Despite these limitations, we were able to capture a broad range of providers’ perspectives from across the country. Furthermore, the richness of qualitative responses from the subset of interviewees highlighted the complexities and nuances that were less evident from the structured survey. Future research that explores both provider and patient experiences qualitatively from participants across the country may provide more insight.

### 5.2 Clinical and public health implications

Providers’ experiences indicate that their pregnant and post‐partum WLHIV patients in the U.S. face a range of complex infant feeding realities and that current guidelines did not meet many of their patients’ needs. These findings are consistent with *Waitt et al*.'s viewpoint piece that thoroughly outlined the biomedical evidence and important gaps in our knowledge of optimal clinical care to support a WLHIV with breastfeeding 32. The tension between current recommendations to avoid breastfeeding and patient desires may continue to inhibit the patient‐provider relationship, which many providers fear may lead some WLHIV to engage in riskier infant feeding behaviours. For instance, some WLHIV may decide to breastfeed in secret rather than oppose provider recommendations directly. This may continue despite the update to Perinatal Guidelines and may need to be explored further. Whether these guidelines change the clinical course given the main message remains the same is uncertain.

## 6 Conclusions

Our study explored providers’ current perspectives and experiences around infant feeding among pregnant and post‐partum WLHIV. Our findings provide important insight to inform professional organizations discussions about public health policy as they consider future directions for infant feeding guidelines among WLHIV. Ultimately, when considering best practices for supporting WLHIV, accounting for the role of how gender, race, culture and HIV‐related stigma intersect with motherhood and infant feeding is paramount.

## Competing interests

ET, MV, CT and JC declare no conflicts of interest.

## Authors’ contributions

JC designed the research study. ET, CT and JC performed the research. JC, MV, ET and CT analysed the data. MV and ET wrote the paper. CT and JC contributed to the draft. All authors read and approved the final manuscript.
